# Enriching the nutritive value of marigold (*Tagetes erecta* L) crop residues as a ruminant feed by lactic acid bacteria during ensilage

**DOI:** 10.1186/s12917-021-02762-8

**Published:** 2021-02-12

**Authors:** Zhijiang Hou, Jianyong Liu, Ming Cai, Yanpei Liu, Lan Mu, Yuee Gao, Metha Wanapat, Bizhi Huang

**Affiliations:** 1 College of Animal Science and Technology, Yunnan Agricultural University, Kunming, 650201 China; 2 Yunnan Academy of Grassland and Animal Science, Kunming, 650212 China; 3 Institute of Alpine Economic Plant, Yunnan Academy of Agricultural Sciences, Lijiang, 674199 China; 4 College of Landscape and Horticulture, Southwest Forestry University, Kunming, 650224 China; 5 Department of Animal Science, Faculty of Agriculture, Khon Kaen University, Tropical Feed Resources Research and Development Center, Khon Kaen, 40002 Thailand

**Keywords:** Biodegradation, Marigold, Terpenes, Volatile organic compounds

## Abstract

**Background:**

Marigold (*Tagetes erecta* L) accounts for over half of the world’s loose flower production, and marigold crop residue (MCR) are abundantly available and should be used as a forage. In this study, MCR from the last commercial flower pickings was ensilaged with lactic acid bacteria (LAB) and the shift in their volatile organic compounds (VOCs) profiles was monitored. Samples were collected at 6 different times during ensilage (3, 6, 9, 12, 15, 30 days) to determine and quantify the VOCs changes using a solid-phase microextraction (SPME) technique and gas chromatography – mass spectrometry (GC-MS).

**Results:**

After 30 days, the caryophyllene and piperitone, which account for 14.7 and 12.1% of total VOCs, decreased by 32.9 and 9.6% respectively, alcohols increased from 2.8 to 8.1%, and the acetic acid content increased by 560%.

**Conclusion:**

We have confirmed LAB can degrade the content of terpenes and enhance the content of alcohols and acids in MCR, which was for the first time on terpene degradation in fodder by ensilage. These results have shed light on our understanding of how to improve fodder odor and to enhance terpene degradation by lactic acid bacteria fermentation.

## Background

Marigold (*Tagetes erecta* L) is one of the most widely cultivated commercial flower crops in the world and accounts for over half of the world’s loose flower production [1]. Since the harvest takes only the flower (used to extract lutein), a large number of marigold residues were randomly discarded. In fact, the crude protein content in the stem of marigold can reach 26.53%, and the content in the leaf is 6.97%, the crude fat content in the stem is nearly double that in the leaf, which can reach 5%. The crude fiber content is 35.09%, while the crude fiber content in the stem is less than 10%, and the stems and leaves are rich in a variety of amino acids [2]. Therefore, marigold crop residue (MCR) should be used as a forage for its high nutritional value and abundantly available. However, studies on the volatile substances in the flowers and leaves of marigolds have indicated that there is a large proportion of various terpenoids, which produces the terpenes of volatile organic compounds (VOCs) would rejected by cattle [3–5].

All domestic mammals have an acute sense of smell, and aroma is one of the most important factors that influencing feed acceptance and intake in cattle [6]. The influence of a specific volatile compound on the final aroma depends on its concentration in fodder and its perception threshold [7–9]. How to reduce the concentrations of main terpenes in total VOC is therefore the key to using MCR as a forage.

Although the VOC terpene level can be reduced through physical and chemical treatments [10, 11], those may cause other issues such as loss of nutritional value, palatability, and safety of such feeds. Another approach to reduce terpene levels is to use biodegradation and biotransformation by microorganisms. Fungi, yeasts, bacteria, cyanobacteria, microalgae, enzymes, plants, and animal cells have all been used in the biodegradation or biotransformation of terpenes [12, 13], however, despite the relative safety of microorganisms and enzymes, only a limited number have been used as feed additives.

In forage processing, ensilage can improve forage palatability and preservation. Ensilage relies mainly on lactic acid bacteria (LAB) fermentation to convert water-soluble carbohydrates into organic acids, and LAB have been widely used as feed additives [14, 15]. The combination of an acidic environment and the microbial fermentation process may synergistically degrade and/or produce new volatiles, and will often produce alcohols and acids which can make the fodder aroma acidic, fragrant, and alcoholic [14, 15] . LAB can also be used in biodegrading and biotransforming terpenes in food fermentation and brewing [16–19].

To the best of our knowledge, there is limited research about the VOC of fodder, and no studies to date on terpene degradation in fodder by ensilage. In order to address this, this study took samples of MCR silage with LAB and used solid-phase microextraction (SPME) and gas chromatography – mass spectrometry (GC-MS) methods to determine and quantify the changes of VOCs over time. The objective of this study was to relate the VOCs changes to ensilage times, and investigate the suitable length of ensilage needed to reduce terpenes while enhancing alcohols and acids to ensure good silage quality.

## Results

Analysis of fresh MCR (CK) showed that the main VOCs were terpenes, which accounted for 63.5% of the 60 VOCs found. Fresh MCR also contained aldehydes (11.35%), ketones (4.61%), esters (3.81%), alcohols (2.8%), alkenes (2.12%), benzenes (1.33%), acids (0.57%), furans (0.56%) phenols (0.48%), and alkanes (0.26%), with other VOCs accounting for the remaining 0.83% (Table. 1).

**Table 1:** Changes in the VOCs (%) of terpenes of MCR ensilage with Lactic acid over time

No	Compounds	CAS	0 day (cK)	3 day	6 day	9 day	12 day	15 day	30 day
		**Terpenes**							
1	(+)-α-pinene	7785-70-8	0.19 ± 0.02A	0.13 ± 0.01AB	0.09 ± 0.05AB	0.09 ± 0.05AB	0.05 ± 0.05AB	ND	0.03 ± 0.03B
2	tricyclo[2.2.1.0(2,6)]heptane, 1,7,7-trimethyl-	508–32-7	0.17 ± 0a	0.09 ± 0.05abc	0.05 ± 0.05bc	0.04 ± 0.04bc	0.13 ± 0.01ab	0.1 ± 0.05abc	ND
3	Linalool	78–70-6	0.57 ± 0.01C	0.81 ± 0.08 BC	0.97 ± 0.11B	0.89 ± 0.04 BC	0.92 ± 0.05 BC	1.05 ± 0.04B	1.42 ± 0.15A
4	terpinen-4-ol	562–74-3	0.47 ± 0.11ab	0.65 ± 0.18a	0.5 ± 0.26ab	0.84 ± 0.18a	0.31 ± 0.31ab	ND	0.01 ± 0.01b
5	Copaene	3856-25-5	0.08 ± 0.04b	0.12 ± 0.06ab	0.1 ± 0.05ab	0.12 ± 0.06ab	0.17 ± 0ab	0.05 ± 0.05b	0.24 ± 0.01a
6	beta-elemene	515–13-9	0.18 ± 0.11a	0.21 ± 0.01a	0.15 ± 0.08a	0.11 ± 0.11a	0.12 ± 0.06a	0.11 ± 0.05a	0.08 ± 0.08a
7	(−)-alpha-gurjunene	489–40-7	0.79 ± 0.04AB	0.84 ± 0.07AB	0.8 ± 0.06AB	0.65 ± 0.04B	0.69 ± 0.1B	0.86 ± 0.08AB	1.13 ± 0.1A
8	Caryophyllene	87–44-5	14.67 ± 0.45A	15.7 ± 0.2A	14.19 ± 0.53A	15.24 ± 0.09A	15.21 ± 0.08A	14.62 ± 0.45A	9.85 ± 0.26B
9	cis-a-bergamotene	18,252–46-5	0.47 ± 0.02a	0.38 ± 0.19a	0.2 ± 0.17a	0.21 ± 0.21a	0.26 ± 0.15a	0.56 ± 0.28a	0.42 ± 0.22a
10	cis-β-farnesene	28,973–97-9	9.46 ± 0.18AB	13.46 ± 1.66A	14.03 ± 1.44A	11.54 ± 0.95AB	11.11 ± 0.96AB	14.04 ± 1.42A	7.97 ± 0.32B
11	2-epi-trans-β-caryophyllene	68,832–35-9	0.24 ± 0.03b	0.69 ± 0.11a	0.6 ± 0.22ab	0.45 ± 0.04ab	0.44 ± 0.04ab	0.53 ± 0.18ab	0.24 ± 0.12b
12	(1e,4e)-germacrene b	15,423–57-1	0.9 ± 0.03 BC	1.93 ± 0.12A	1.49 ± 0.13AB	1.48 ± 0.08AB	1.6 ± 0.15AB	0.93 ± 0.23 BC	0.26 ± 0.26C
13	(1 s,2e,6e,10r)-3,7,11,11-tetramethylbicyclo[8.1.0]undeca-2,6-diene	24,703–35-3	0.6 ± 0.06A	0.32 ± 0.07B	0.11 ± 0.02C	ND	ND	ND	ND
14	ß-longipinene	41,432–70-6	0.24 ± 0.09b	0.46 ± 0.06ab	0.39 ± 0.1ab	0.56 ± 0.19ab	0.6 ± 0.12a	0.25 ± 0.05ab	0.29 ± 0.06ab
15	2-carene	554–61-0	0.17 ± 0.02A	ND	ND	ND	ND	ND	ND
16	naphthalene, 1,2,3,5,6,7,8,8a-octahydro-1,8a-dimethyl-7-(1-methylethenyl)-, [1 s-(1a,7a,8aa)]-	10,219–75-7	0.09 ± 0.09b	0.33 ± 0.02a	0.18 ± 0.09ab	0.23 ± 0.04av	0.22 ± 0.06ab	0.19 ± 0.05ab	0.18 ± 0.05ab
17	(−)-germacrene-d	317,819–80-0	ND	1.95 ± 0.18A	0.97 ± 0.31ABC	1.43 ± 0.49AB	1.58 ± 0.36AB	0.35 ± 0.14 BC	0.8 ± 0.04ABC
18	(−)-spathulenol	77,171–55-2	ND	0.13 ± .0.01a	0.15 ± .0.02a	0.16 ± .0.01a	0.15 ± 0.02a	0.13 ± .0.03a	0.15 ± 0.02a
19	Junenol	472–07-1	ND	0.2 ± 0.03CD	0.21 ± 0.01CD	0.42 ± 0.03 BC	0.45 ± 0.01B	0.48 ± 0.01B	1.1 ± 0.13A
20	Carveol	99–48-9	ND	ND	ND	ND	0.37 ± 0.19ab	0.17 ± 0.17ab	0.07 ± 0.07b
21	Isothujol	7712-79-0	ND	ND	ND	ND	ND	0.05 ± 0.05B	0.34 ± 0.01A
23	(−)-α-thujone	33,766–30-2	ND	ND	ND	ND	ND	ND	0.07 ± 0.01A
24	(−)-.β.-bourbonene	5208-59-3	ND	ND	ND	ND	ND	ND	0.14 ± 0.01A
25	(s)-β-bisabolene	495–61-4	ND	ND	ND	ND	ND	ND	0.14 ± 0.02A
26	Nerolidol	7212-44-4	ND	ND	ND	ND	ND	ND	0.89 ± 0.11A
27	3,7,11,15-tetramethyl-2-hexadecen-1-ol	102,608–53-7	ND	ND	ND	ND	ND	ND	0.24 ± 0.07A
28	Isoneral	72,203–97-5	ND	ND	ND	ND	ND	0.15 ± 0.01B	0.27 ± 0.03A
29	(±)-trans-nerolidol	40,716–66-3	ND	ND	ND	ND	ND	ND	0.88 ± 0.07A
30	bicyclo[3.1.1] heptane, 6,6-dimethyl-2-methylene-, (1 s)-	18,172–67-3	3.01 ± 0.27A	0.96 ± 0.15 BC	1.18 ± 0.07 BC	1 ± 0.2 BC	1.24 ± 0.08 BC	1.21 ± 0.04 BC	0.56 ± 0.05 BC
31	terpinolene	586–62-9	8.24 ± 0.25A	7.72 ± 0.2AB	7.15 ± 0.18ABC	6.8 ± 0.15 BC	7.18 ± 0.59ABC	6.27 ± 0.05C	4.62 ± 0.15D
32	d-limonene	5989-27-5	5.56 ± 0.14A	3.55 ± 0.13B	3.49 ± 0.24 BC	3.32 ± 0.12 BC	3.44 ± 0.22 BC	3.3 ± 0.05 BC	2.57 ± 0.36C
33	ß-ocimene	13,877–91-3	4.92 ± 0.31A	4.2 ± 0.23A	4.07 ± 0.24A	3.98 ± 0.27A	4.43 ± 0.32A	4.21 ± 0.22A	2.38 ± 0.27B
34	Piperitone	89–81-6	12.17 ± 0.11a	9.93 ± 0.47b	10.08 ± 0.68b	10.76 ± 0.32ab	10.16 ± 0.31b	10.51 ± 0.24b	10.1 ± 0.7b
35	Sabinene	3387-41-5	0.31 ± 0.06A	ND	ND	ND	ND	ND	ND

*CAS* Chemical Abstract Service, *ND* Not detected, experiments were performed in triplicate and shown are the means ± S.D., different uppercase letters in the same column correspond to significance at the 0.01 level, and different lowercase letters correspond to a significant difference at the 0.05 level (Duncan’s new multiple range test). The same as Table 2

**Fig. 1:**
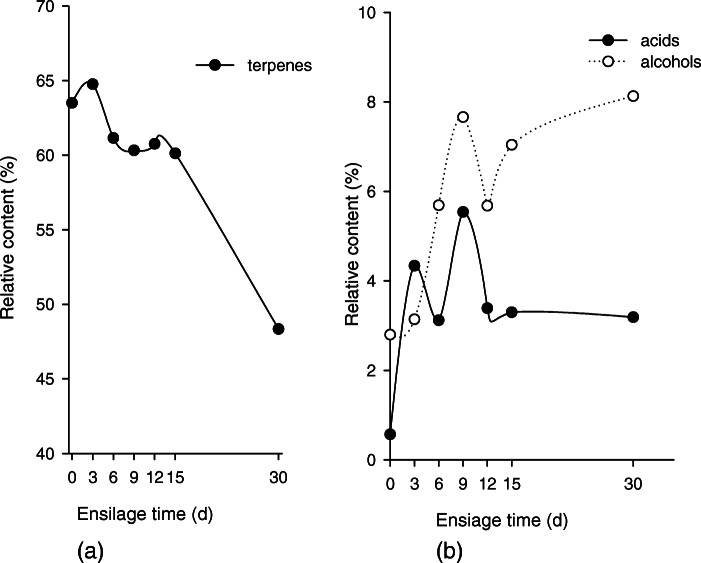
Changes in terpene (**a**) and alcohols and acids (**b**) content in MCR during ensilage

**Fig. 2:**
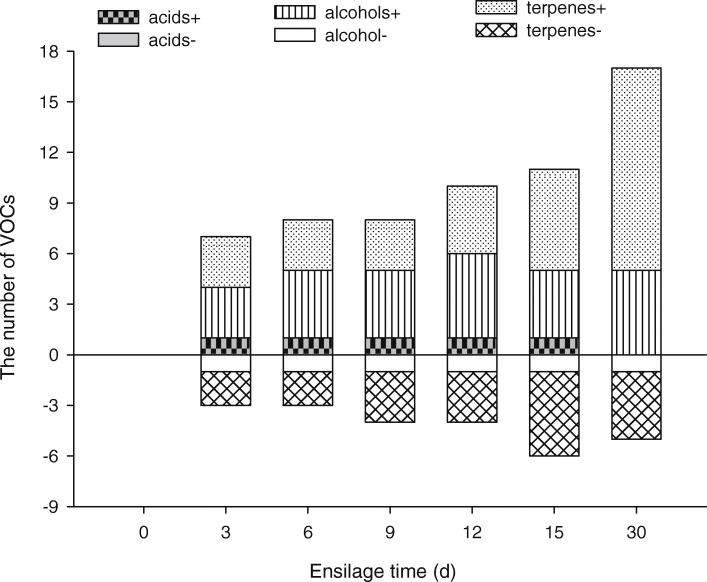
Changes in number of main VOCs in MCR during ensilage. + represents the VOCs that appeared, − represents the VOCs that disappeared

The differences in terpene levels between fresh MCR and MCR at different silage treatment times are shown in Table. 1. The levels of caryophyllene, the main VOC which accounted for 14.67% of total VOCs, were relatively stable on days 3, 6, 9, 12, 15 (*P* > 0.01), but on day 30 had decreased by 32.86% (*P* < 0.01). Another main VOC, piperitone, which accounted for 12.17% of total VOCs, declined on days 3, 6, 9, 12, 15, and 30 by 18.41% (*P* < 0.05), 17.17% (P < 0.05), 11.59% (P < 0.05) and 16.52% (P < 0.05), 13.64% (P < 0.05), and 17.01% (P < 0.05) respectively. Compared with the fresh MCR group, levels of caryophyllene, the most abundant constituent of total VOCs, decreased noticeably on day 30 by 32.86% (*P* < 0.01), but not at any other time during ensilage. While the levels of some terpenes that accounted for a small amount of total VOCs increased with silage time, overall the total amount of terpene VOCs decreased by 33.87% after 30 days (Fig. 1a). As ensilage progressed, not only did some terpenes disappear, new terpenes were produced, among those, 12 of which are exclusively found after ensilage. Many of the newly generated VOCs were produced on the 30th day of ensilage (Fig. 2).

The alcohol levels of fresh MCR and MCR at different ensilage times are shown in Table 2. After 30 days of ensilage, the content of the original alcohols did not significantly change, and only one of the alcohols disappeared. This study found that 6 alcohols appeared after ensilage (Fig. 2). Compared with other alcohols, (3-Methyl-oxiran-2-yl)-methanol was the most abundant alcohol found in silage, accounting for 4.14% of total VOCs. After 30 days’ ensilage the total amount of alcohols in VOCs increased from 2.8 to 8.13% (Fig. 1 b).

**Table 2:** Changes in the VOCs (%) of alcohols and acids of MCR ensilage with Lactic acid over time

No	Compounds	CAS	0 day (cK)	3 day	6 day	9 day	12 day	15 day	30 day
**Alcohols**									
36	(2r,3r)-(−)-2,3-butanediol	24,347–58-8	0.04 ± 0.04a	0.38 ± 0.26a	0.21 ± 0.13a	0.56 ± 0.34a	0.8 ± 0.11a	0.25 ± 0.05a	0.21 ± 0.02a
37	leaf alcohol	928–96-1	1.42 ± 0.19A	0.46 ± 0.16B	0.27 ± 0.04B	0.32 ± 0.06B	0.33 ± 0.07B	0.13 ± 0.07B	0.09 ± 0.09B
38	1-hexanol	111–27-3	0.42 ± 0.07A	0.21 ± 0.05B	0.18 ± 0.02B	0.18 ± 0.01B	0.17 ± 0.02B	0.15 ± 0.01B	0.04 ± 0.04B
39	9-oxabicyclo[6.1.0]nonan-4-ol	69,853–85-6	0.03 ± 0.02	ND	ND	ND	ND	ND	ND
40	phenylethyl alcohol	60–12-8	0.49 ± 0.27a	0.49 ± 0.39a	0.33 ± 0.33a	0.58 ± 0.19a	0.66 ± 0.14a	1.02 ± 0.15a	0.36 ± 0.19a
41	trans-chrysanthenol	38,043–83-3	0.15 ± 0.01C	0.25 ± 0.02B	0.27 ± 0.02B	0.27 ± 0.01B	0.27 ± 0.01B	0.31 ± 0.01B	0.39 ± 0.02A
42	2-(4-methylphenyl)propan-2-ol	1197-01-9	0.25 ± 0.02 a	0.3 ± 0.03a	0.37 ± 0.05a	0.34 ± 0.05a	0.35 ± 0.15a	0.34 ± 0.21a	0.62 ± 0.08b
43	(3-methyl-oxiran-2-yl)-methanol	872–38-8	ND	0.48 ± 0.22CD	2.82 ± 0.56AB	2.18 ± 0.59ABC	1.7 ± 0.68BCD	3.25 ± 0.1AB	4.14 ± 0.43A
44	3-methylcyclopentane-1,2-diol	27,583–37-5	ND	ND	ND	ND	0.11 ± 0.06A	ND	ND
45	benzyl alcohol	100–51-6	ND	0.38 ± 0.15a	0.44 ± 0.2a	0.27 ± 0.02ab	0.23 ± 0.03ab	0.26 ± 0.05ab	0.38 ± 0.07a
46	(+)-maaliol	527–90-2	ND	0.19 ± 0.02 BC	0.22 ± 0.05 BC	0.32 ± 0.04B	0.39 ± 0.04B	0.33 ± 0.03B	0.65 ± 0.11A
47	2-butanol	15,892–23-6	ND	ND	0.58 ± 0.04a	2.64 ± 2.26a	0.56 ± 0.06a	1 ± 0.17a	1.02 ± 0.26a
48	3-methylcyclopentane-1,2-diol	27,583–37-5	ND	ND	ND	ND	0.11 ± 0.06A	ND	ND
49	decan-1-ol	112–30-1	ND	ND	ND	ND	ND	ND	0.23 ± 0.01A
**Acids**									
50	acetic acid	64–19-7	0.57 ± 0.01b	4.18 ± 1.40a	2.98 ± 1.03ab	5.37 ± 1.50a	3.17 ± 0.33ab	3.27 ± 0.43a	3.19 ± 0.37ab
51	cyclohexanebutanoic acid	4441-63-8	ND	0.16 ± 0.09a	0.14 ± 0.09a	0.17 ± 0.09a	0.22 ± 0.08a	0.03 ± 0.01a	ND

Only two acids, cyclohexanebutanoic acid and acetic acid, were present during the whole fermentation process (Table. 2). The cyclohexanebutanoic acid content was very low, and was detected on only the third to fifteenth day of ensilage, while the acetic acid accounted for 0.57% of total VOCs in fresh MCR, but increased by 560% (*P* < 0.05) after ensilage.

Ensilage not only changed the levels of terpenes, alcohols, and acids in the MCR, but also changed the volatile profile of other quantitative and qualitative compounds (Table 3). On day 30, declines in the total aldehyde (14.45%) and benzene (53.38%) VOCs, and increases in the total VOCs of esters (60.63%), phenols (454.17%), alkanes (434.62%), ketones (47.36%), alkenes (74.06%), furans (103.57%), and miscellaneous compounds (174.70%) were observed.

**Table 3:** Changes in the VOCs (%) of others of MCR ensilage with Lactic acid over time

No	Compounds	0 day (cK)	3 day	6 day	9 day	12 day	15 day	30 day
1	Aldehydes	11.35	6.23	6.61	7.05	6.42	7.48	9.71
2	Phenols	0.48	0.88	1.16	1.31	1.6	1.81	2.66
3	ketones	4.61	5.43	5.57	6.02	5.87	5.84	6.13
4	Esters	3.81	6.8	7.01	7.65	5.77	6.11	6.12
5	alkanes	0.26	0.47	0.64	0.41	2.05	0.7	1.39
6	alkenes	2.12	1.1	0.99	1.03	0.7	0.44	3.71
7	benzenes	1.33	1.2	1.02	0.94	0.95	0.78	0.62
8	Furans	0.56	0.68	0.62	0.64	0.65	0.65	1.14
9	miscellaneous	0.83	0.77	0.76	0.86	1.69	2.07	2.05

## Discussion

Caryophyllene, piperitone, cis-β-farnesene, and terpinolene found in this study represented the major components of the essential oil of marigold leaves and flowers as well [4, 20]. Terpene was the major component of VOCs in marigold flowers which consistent with a previous report and may suggest that high terpene content of VOCs could be the main reason for MCRs’ pungent taste [21].

This study describes the effects of LAB on the biotransformation of VOCs from MCR. These results showed LAB mediated degradation of some terpenes, which agreed with those of a previous study conducted by Figueiredo et al. [22] who found that terpenes in red clover forages decreased greatly after ensilage. Park et al. [19] also found that LAB significantly reduced the terpene content of blueberry juice, including a 92% reduction in vitispirane. The causes of terpene reduction were not fully known, but could involve oxidation to secondary products, glycoside hydrolysis, or ester conversion, as well as isomerization and / or interconversion of some monoterpenols [13, 23–25].

However, not all terpene levels were changed. This study has shown that some of the main occurring terpenes were not degraded by LAB, similar to the study of Belviso et al. [26] which showed that while alpha-campholenal can be completely degraded in LAB cultures, alpha-pinene, alpha-terpineol, beta-myrcene, and myrtenal did not degrade at all. Liu et al. [18] reported that some terpenes might be difficult to hydrolyze because their precursors were in the bound form. This could mean that some of these terpenoids present may be in their bound form at the end of the ensilage, or this might be due to enzymatic hydrolysis by glycosidases from microorganisms being limited under the specific conditions found during fermentation.

An explanation for this late silage degradation of terpenes may be that glycoside precursors were mainly released by acid hydrolysis, a process that occurred slowly [8]. Therefore, terpene levels changing at different times during ensilage could be a result of the different levels of glycoside resistance to acid hydrolysis.

According to the current literature, total content of terpene in forage can be reduced by ensilage, but there were still a small amount of terpenes increased [22], which is consistent with our results. Similarly, total terpene content declined when LAB was used to ferment berry juice [19], but the total terpene content increased when pomegranate juice was fermented [17]. There is a paucity of information regarding terpene biodegradation by LAB, and studies have shown that terpene biodegradation varies across different species and strains of microorganisms, including LAB. The results from this study have provided preliminary information for future studies on terpene biodegradation in MCR fermentation.

Belviso et al. [26] found that LAB cultures can completely degrade alpha-campholenal and form a new monoterpenoid in 48 h. Although terpenes are formally composed of one biosynthetic unit, the fact that they can be biotransformed by mechanisms including hydration, isomerization, dehydrogenation, conjugation, oxidation, reduction, decarboxylation, and β-oxidation, means that multiple structures can be produced [2, 13]. Microorganisms that promote the biological transformation of terpenes include bacteria, fungi, and yeast. These microorganisms can transform the original terpenes into new ones and other substances via various biotransformation reactions [2, 13]. Thus, LAB is responsible for both the degradation of terpenes and the production of the new terpenoidic metabolites. The terpene biotransformation mechanisms of LAB are not well established. Although there have been some reports about the biotransformation activities of LAB during juice and pickle fermentation [16, 19], it is difficult to infer the complex relationships between them based on the changes in either the final amount of terpenes or in the kinetics, since there are many other compounds that could interact with terpenes or influence the metabolic behavior of LAB.

These results are consistent with those of Figueiredo et al. [22] who also found that the levels of original alcohols in red clover did not significantly change after ensilage and that some alcohols disappeared.

Wide variations in alcohol levels have been observed for different forage, with comparable or lower concentrations seen in corn, alfalfa, cereal and red clover silages [3, 22, 27, 28]. Current research suggests that a large amount of alcohol is produced during ensilage and that the volatile content of ethanol in corn silage is up to 70% of total VOCs [3, 27, 28], however, in this study, no ethanol was detected at any stage. Except for ethanol, there is a lack of data on alcohols in silage which are probably generated by amino acid catabolism or by the reduction of aldehydes and ketones [16].

High ethanol contents have been observed in high-dry-matter grass silages due to their high content of fermentable carbohydrates. Low carbohydrate legume forages do not produce more ethanol during the ensiling process [22, 28]. Low fermentable carbohydrate may be the main reason for the absence of ethanol during the MCR fermentation process, while silage quality is not measured by the production of large quantities of ethanol, which can adversely affect both the environment and the animals themselves [3, 27, 28].

Acetic acid is the most important organic acid in silage, affects its quality, and is known to possess a sour odor [29]. Acetic acid accumulation depends on substrate supply and the sugar metabolism of the starter culture [30]. In fat metabolism during ensilage, LAB could degrade fatty acids to produce short-chain fatty acids such as butyric acid, acetic acid, butyric acid, and caprylic acid. Goswami et al. [31] found that acetic acid and butyric acid concentrations were significantly increased during the fermentation of horse gram by *Lactobacillus plantarum* (NRRL-B 4496) and *Lactobacillus plantarum* (NCDO 1133), indicating that these two strains can effectively metabolize fatty acids to produce short chain fatty acids.

As more acid could be produced in other silage and food fermentation processes, the detection of only two acids in this experiment make this study differ from the rest of the current literature. Since the acids produced by LAB species are strain-dependent [32], further research is needed on the importance of organic acids to silage quality.

Other VOCs, even at lower concentrations, might considerably influence animal acceptance of forage [33]. In this study, it was not possible to elucidate a clear and definite relationship between MCR ensilage with LAB and VOC biotransformation or to distinguish between the effects of the various VOCs observed. Hence, more research on this specific relationship should be conducted.

## Conclusions

This work presents the first investigation of the biotransformation of VOCs in MCR by LAB ensilage. The results reported in this study show that during ensilage, LAB influences type and levels of VOCs. Compared with the fresh MCR group, the main VOCs caryophyllene and piperitone were decreased by 32.9 and 17.0%, respectively after 30 days of ensilage, while the content of alcohols increased from 2.8 to 8.1%, and the acetic acid content increased by 560%. The findings of this study should form the base foundation for future studies leading to elucidate more suitable LAB strains and their optimal environmental conditions, including concentration, pH and temperature which would allow for operations to be scaled-up. Meanwhile, these results have shed light on our understanding of how to improve fodder odor and to enhance terpene degradation by lactic acid bacteria fermentation.

## Methods

### Plant materials and bacterial strains

MCR was obtained from Tengchong city, Yunnan Province, at the end of September 2019 after the last commercial flowers had been picked while the stems and leaves were still green and fresh. The MCR was manually mowed leaving 2–3 cm of stubble and air-dried away from light until moisture levels had dropped to about 75%. *Lactobacillus plantarum* LP-115 (Danisco USA Inc., Madison, WI, USA) was used in the fermentation of MCR.

### Silage preparation

The MCR was chopped into pieces approximately 3 cm in length using a forage cutter (Lingong Machinery, Shandong, China), thoroughly mixed and either treated with LAB or left untreated (control). A total of 18 silage and 3 control replicates were set up. On the first day of the experiment, 5 mg/kg of *Lactobacillus plantarum*, containing lactic acid bacteria (LAB) at (1.0 × 10^5^) colony forming unit (cfu)/g, was added to the fresh MCR as per manufacturer’s instructions. To produce silage, the MCR was compressed into a 1 L polyethylene bag silo (Beijing meat processing company, Beijing) and in order to ensure an anaerobic fermentation environment, all bags were sealed with a vacuum packer (Beijing Keyoujia, Beijing) and stored indoors in the dark for 30 days at 25 °C. Three samples were taken from the control group and silage treatments at 3, 6, 9, 12, 15 and 30 days and frozen at − 20 °C prior to analysis of VOCs.

### SPME experimental conditions

After the sample was melted, 3 g of MCR sample was put into a 20 mL Agilent crimp-top headspace vial, and heated in a 60 °C water bath to allow the aroma substances in the extraction bottle to reach equilibrium. After 5 min, the aged extraction head was inserted and extraction at 60 °C for 30 min was performed before GC-MS analysis.

### GC-MS analysis

GC-MS was used to analyze the VOCs from MCR. A TRACE1310/ISQ7000 mass selective detector (ThermoFisher) was used in conjunction with a TG-5MS column (30 m*0.25 mm*0.25um; ThermoFisher). Operation conditions were as follows: Set injection to splitless mode for 5 min at 250 °C. Helium flow rate, 1.0 mL/min. Temperature programing: 40 °C for 2 min then 4 °C/min to 160 °C for 4 min and finally ramped to 250 °C at 15 °C/min and maintained for 2 min. The temperature of ion source was set to 230 °C and the inlet line temperature was set at 250 °C. The MS detector operated in positive electron ionization (EI+) mode at 70 eV under a mass scan range of 35–450 amu (m/z). VOCs were initially identified by comparison with the mass spectra data registered in the National Institute of Standards and Technology database (NIST 11) (Avila-Sosaet al., 2010), and identity was further ascertained based on the probable percentage of the three candidate components provided by GC-MS. The relative percentage of each component was calculated using the total percentage of peak area which was expressed as a percentage of the sum peak area of all identified compounds.

### Statistical analysis

SPSS 19. 0 Statistical software was used to perform analysis of variance, and multiple comparisons using Duncan’s method (*P* = 0.01 and *P* = 0.05). Mapping was performed using Sigmaplot 10.0.

## Data Availability

The datasets used and analysed during the current study are available from the corresponding author on reasonable request.

## Abbreviations

CAS: Chemical Abstract Service
GC-MS: Gas chromatography – mass spectrometry
LAB: Lactic acid bacteria
MCR: Marigold crop residue
SPME: Solid-phase microextraction technique
VOCs: Volatile organic compounds
